# Unlocking gender dynamics in food and nutrition security in Ghana: assessing dietary diversity, food security, and crop diversification among cocoa household heads in the Juaboso-Bia cocoa landscape

**DOI:** 10.1186/s12889-024-18204-7

**Published:** 2024-04-08

**Authors:** Michael Batame

**Affiliations:** 1 Warnell School of Forestry and Natural Resources, University of Georgia, Athens, GA 30602 USA; 2 Department of Natural Resources Management, Faculty of Geoinformation Science and Earth Observation, ITC, University of Twente, Enschede, The Netherlands

**Keywords:** Nutrition, Food security, Mixed research methods, Dietary diversity score, Crop diversification, Rural cocoa household

## Abstract

**Background:**

Malnutrition is a worldwide problem that impacts every country, affecting one in three individuals, including Ghana. According to estimates from the Food and Agriculture Organization (FAO), 690 million people were undernourished globally in 2019. Malnutrition is no exception in rural cocoa communities in Ghana. The expansion of cocoa is causing food insecurity and low dietary diversity in most cocoa-growing areas. There is little information on the food security status and dietary diversity among male-headed and female-headed cocoa households in the Juaboso-Bia cocoa landscape. Thus, this study sought to explore the dietary diversity distribution, food security status, sources of staple food, food unavailability periods, food production status, themes contributing to low dietary diversity and food insecurity, and perception of the impact of cocoa expansion on crop diversification among male-headed and female-headed cocoa households in the study area.

**Methods:**

Both quantitative and qualitative research methods were employed to address the research questions. The study collected 200 semi-structured questionnaire data and 14 in-depth interview data from cocoa household heads in the Juaboso-Bia landscape. The survey data was cleaned and analysis, such as household dietary diversity status, food security status, and binary logistic regression were performed in the Statistical Package for Social Scientists (SPSS). The in-depth interviews were analyzed using thematic analysis.

**Results:**

Through this study, it was revealed that 62.8% of the male-headed cocoa households had medium to high dietary diversity compared to their female counterparts 39.3%. About 47.9% of the male-headed households were food secure than the female-headed households (29.1%). Moreover, the months that both male and female-headed households recalled facing severe food unavailability were July and June. In addition, climate change/variability, unavailable lands, poverty, large household size, and gender stereotypes were themes promoting low dietary diversity and food insecurity among male and female-headed households. Furthermore, sex, total household income, and cropland conversion to cocoa were the variables influencing household heads’ perception of the impact of cocoa expansion on crop diversification.

**Conclusions:**

The study showed that the male-headed cocoa households had high dietary diversity and were food secure than the female-headed cocoa households, lending credence to the conceptual framework applied in this study. There is a significant relationship between household head gender and food security status. Therefore, this study recommends the following interventions to improve dietary diversity and food security among male and female-headed cocoa households: raise awareness about the need for diverse diets and provide practical information on how to incorporate a greater variety of food groups into their daily meals; and promote gender equity and inclusivity in food security interventions. Future research could investigate how gender mainstreaming policies in agriculture have helped empower and improve the food security of female-headed households in Ghana.

**Supplementary Information:**

The online version contains supplementary material available at 10.1186/s12889-024-18204-7.

## Introduction

Globally, one in three people suffers from malnutrition [1]. Approximately 690 million people worldwide were undernourished in 2019, according to the Food and Agriculture Organization (FAO) estimate [2]. In emerging nations, malnutrition can become worse, particularly in rural areas where the poorest people are most concentrated [3]. Malnutrition has many different root causes, but it is more prevalent in areas where there is evidence of rising food insecurity, which is frequently linked to the occurrence of climate change events [2]. For instance, in southern Africa, erratic rainfall that is typically linked to climate change impacts farmers’ agricultural yields, which in turn affects their food security and nutrition [4].

Despite major improvements in food and nutrition security over the past few decades, particularly in sub-Saharan Africa (SSA) and South Asia, the prevalence of undernutrition is still high [1]. In Ghana, there have been numerous attempts to reduce the rate of malnutrition, and these efforts have steadily improved [5]. However, Ghana still struggles with the issue of malnutrition, which has contributed to 50% of child fatalities [6]. Malnutrition in Ghana is largely attributed to high intake of carbohydrate-rich food, namely cassava, maize, and rice, and inadequate food intake rich in vitamins and proteins, for example, meat, eggs, milk products, legumes, and fruits [7].

The Juaboso-Bia cocoa landscape is a rural district where its inhabitants are predominantly cocoa farmers and also engage in subsistence farming [8]. Many cocoa households in this area have expanded their cocoa production intending to increase their household income and secure their food and nutrition security [8–10]. This cocoa production expansion ends up displacing food croplands hence exacerbating households’ food and nutrition insecurity in this cocoa rural district [8]. Moreover, [11] argued that food availability in rural places depends on the environment’s natural resources and the ability to produce food through agriculture, more specifically, the availability of a variety of agricultural items for self-consumption raises the dietary diversity of households in agricultural production areas. The study by [7] showed that in cocoa-growing areas, cocoa households usually consume staple foods (roots, tubers, oil, and small dried fish) that are not sufficient in micronutrients to counter undernutrition. Malnutrition occurs in Ghana’s cocoa regions as a result of the majority of cocoa households’ undiversified diets (consuming less than 5 food groups daily), which also stunts their growth, especially among women [7].

Food and nutrition security has several facets, and one of them is the quality of diets, and the dietary diversity score (DDS) is a well-known numerical indicator of diet quality [1, 12–14]. Dietary diversity offers information on the availability of a variety of foods in a household and can be used as a proxy for a person’s diet’s nutrient sufficiency [15]. For example, an increase in a person’s dietary diversity score is related to an increase in diet nutrient sufficiency [16]. Healthy eating is defined as consuming a varied, well-balanced diet that includes foods high in vitamins and minerals, as well as fruits, vegetables, and fresh, natural meals [15]. It also requires practicing healthy eating practices and engaging in activities that advance one’s physical and mental health [7, 17, 18]. The number of various foods or food groups in a diet is used to calculate dietary diversity. A concept known as household dietary diversity (HDD) is used to gauge a household’s capacity to pay for a range of foods throughout a given period [2]. A common tool for evaluating food consumption, including the degree of variety of foods that a household has access to, is the Household Dietary Diversity Score (HDDS), which is generated from the dietary diversity questionnaire [2]. This HDDS is limited by factors, such as income, land size, household size, source of staple food, and age and education level of the household [3, 8, 15, 19, 20].

There have been extensive studies conducted on cocoa production and its impact on household food security in Ghana. For instance, [21] examined food security among male and female cocoa households in Wassa Amenfi West District, Western North Region of Ghana. The study used the household dietary diversity score to measure the food security status of cocoa households. The study found that the female-headed cocoa households were more food insecure than the male-headed cocoa households. Also, [8] investigated how farmers convert land use from food crops to cocoa, as well as the primary factors that influence the transition. According to their findings, food cropland has been drastically reduced as a result of cocoa expansion, affecting farmers’ food self-sufficiency. Again, [22] discovered that households with lower dietary diversity had lower production diversity, while households with higher dietary diversity experienced higher production diversity in Indonesia. Dietary diversity was primarily loss due to decreased consumption of healthful food groups, such as fruits, vegetables, legumes, and fish. Several food groups, such as dairy, meats, and eggs, have been heavily consumed over the years and are good components of nutritious diets. Furthermore, [23] revealed that gender, level of education, source of food, and wealth of the head of household were the factors influencing dietary diversity in urban households in Accra, Ghana. Their studies also found that vegetables were most consumed followed by cereal-based and grain products.

However, these studies did not provide insight into dietary diversity distribution among male and female-headed households, months in which food insecurity occurs, accessibility to sources of stable food, crop diversification, and factors accounting for low dietary diversity. Thus, the Juaboso-Bia cocoa landscape in Ghana has no information on the distribution of dietary diversity and food security patterns among male and female-headed cocoa households, and its associated factors. Dietary diversity is necessary to be explored since it has multifaceted reasons, such as health, nutrition, and socio-economic benefits. Encouraging individuals and communities to embrace a diverse and balanced diet is a key strategy for addressing nutritional challenges and promoting holistic well-being [10, 17].

To address these gaps, this study, therefore, examined the dietary diversity distribution and food security status among male and female-headed cocoa households and its influencing factors in the Juaboso-Bia cocoa landscape. The hypotheses for this study were: H_0_ = there is no significant relationship between food security status and gender of the household head, and H_1_ = there is a significant relationship between food security status and gender of the household head. Research questions explored for answers were: 1. How is the dietary diversity and food security status in male and female-headed cocoa households distributed?; 2. Where do the male and female-headed cocoa households obtain their staple food?; 3. What is the food production status among male and female-headed cocoa households?; 4. Which months do the male and female-headed cocoa households experience low consumption of dietary diversity?; 5. What are the contributing themes for the low dietary diversity and food insecurity in the male and female-headed cocoa households’?; and 6. How do cocoa household heads perceive the impact of cocoa expansion on crop diversification in the Juaboso-Bia landscape? These answers are crucial for comprehending dietary diversity across male and female-headed households within the highest levels of food insecurity, allowing policymakers to properly implement tailored nutrition interventions as well as informing and providing feedback to development activities. This study’s results will contribute to a large body of knowledge on the nexus of cocoa production, food security, and dietary diversity.

### Literature review

This sub-section explores studies on food security and dietary diversity as well as their determinant factors.

#### Food security and dietary diversity

Many rural people experience food and nutrition insecurity [2]. The study by [2] sought to identify the factors that influence the Household Dietary Diversity Score (HDDS) in the rural area of the Paute River Basin, Azuay Province, Ecuador. A stratified random sampling technique with proportional affixation was employed to determine the sample size of 383 surveys. The HDDS was used to quantify dietary diversity using 12 food groups over a 7-day recall period: cereals, fruits, sugar/honey, eggs, meat and eggs, legumes or grains, vegetables, oils/fats, milk and dairy products, meats, miscellaneous, fish, and shellfish. The association between the HDDS and sociodemographic factors was evaluated using a Poisson regression model. The findings indicated that carbohydrates, fruits, roots and tubers, and sugar/honey were the most often consumed food groups among those evaluated. Furthermore, the predictive model’s most effective drivers of the HDDS were housing size, household size, per capita food expenditure, area under cultivation, educational attainment, and the head of household’s marital status.

Furthermore, [24] examined the determinants of dietary diversity score for rural households of Uttar Pradesh State in India using 248 surveyed households through stratified random sampling. The study assessed household dietary diversity score and individual dietary diversity score, using 12 food groups and 9 food groups developed by FAO, respectively. The results indicated that cereals, tubers, oils and fats, spices, and condiments are the most often consumed foods in the household. When it comes to dietary diversity, female-headed households scored lower than male-headed households. There was a slight positive correlation found between the household dietary diversity score and the head of the household’s nutritional knowledge, awareness, attitude, and educational status.

Ref. [17] explored the determinants of rural household dietary diversity in the Amatole and Nyandeni districts in South Africa. A household cross-sectional survey data from 181 rural communities was collected, that is 100 from Amatole and 81 from Nyandeni. The household dietary diversity score was employed to calculate the dietary diversity of the respondents using 12 food groups over a 24-hour dietary recall. The results showed that these food groups were largely consumed: sugars (16%), condiments (16%), oils (12%), potatoes (12%), grains (11%), and beans/peas (9%), whereas these food groups: milk (6%), vegetables (5%), eggs (4%), meats (3%), fruits (3%) and fish (2%) were least consumed. The multinomial logistic regression model predicted that participation in irrigation schemes, gender, education, income, access to home gardens, and ownership of small livestock attainment positively influenced high dietary diversity.

Ref. [25] examined the factors that determine household food security in the Sekyere-Afram Plains District of Ghana. The authors randomly selected 4 communities and interviewed 25 households per community, so in total interviewed 100 households. The USDA Household Food Security Scale was utilized to estimate the food security status, and a binary logistic regression was used to predict the determinant of food security. Household size, farm size, off-farm income, credit access, and marital status were positively related to food security.

Ref. [3] analyzed dietary diversity in rural areas, especially in the case of indigenous communities in Sierra Tarahumara, Mexico employing household dietary diversity score. The 123 survey data was collected in February and March 2015. Cereals (100%) were the food group most consumed by the study sample, followed by legumes or nuts (96.7%), eggs (78.9%), sugar/honey (78%), and oils/fats (77.2%). The best variables that positively influenced dietary diversity were food expenditure per capita, part-time work, the Prospera program, and the head of household’s marital status.

Moreover, [1] predicted the variables that influence dietary diversity and the potential role of men in improving household nutrition in Tanzania. The study used a multi-stage sampling technique, which comprised 4 stages: (i) Bani and Mbarali were purposively selected because of their prevalence of poverty and malnutrition; (ii) From the list generated by the Competitive African Rice Initiative (CARI) program administrators and District Agriculture Irrigation and Cooperatives Officers (DAICOs), 20 villages were chosen at random; and a proportionate random sample of the targeted households based on the number of households in the village taking part in the CARI program, yielding 204 respondents in total. The authors utilized cross-sectional survey data from the Bani and Mbarali Districts. There were 101 and 103 participants from Bani and Mbarali Districts, respectively. The results indicate that the foods that are most frequently consumed in the home are cereals, vegetables, oils and fats, spices, condiments, and beverages. Dietary diversity was lower in households led by women than it was in households headed by men. From mid-November to March, male-headed households experience food shortages. Gender and education of the household head and food preparation and nutrition training were significant determinants of household dietary diversity.

This study by [26] looked at the connections between household income, credit availability, and dietary diversification. Dietary diversity was calculated using the Food Diversity Index and Food Consumption Score. The authors used 5779 and 8312 household survey data from the 4th and 5th rounds of the Ghana Living Standards Survey. The findings revealed that access to credit/income was found to be positively correlated with dietary diversity since having an income greatly influences the food types households consume in Ghana.

Ref. [19] study aimed to investigate, from a gender viewpoint, the dietary diversity among Nigerian rural households. The Simpson Diversity Index, cross-tabulation, and Oaxaca-Blinder decomposition were used to analyze data from the Living Standard Measurement Survey-Integrated Survey on Agriculture 2016. The results unveiled that the score for dietary diversity was somewhat higher in households headed by women than in households headed by men. In households led by women, food share expenditure was highest for fish and seafood, whereas in homes headed by men, it was highest for cereals. Households headed by males aged 31 to 40 and comprising a minimum of 16 members had low dietary diversity, whereas households headed by females aged 51 to 60 and comprising 11 to 15 members displayed the highest level of food diversity.

Ref. [27] argued that before the cocoa harvest, many households in West Africa that cultivate cocoa go through a “lean season,” which makes them susceptible to a range of stressful situations, chief among them being food insecurity. The author investigated the effects of income distribution and intra-household dynamics on household resilience during the lean season, primarily using qualitative data from Côte d’Ivoire. Also, the [28] study’s goal was to examine the factors that influence household dietary diversity and consumption patterns in the Yayu biosphere reserve in southwest Ethiopia. A cross-sectional survey including 183 randomly chosen households was carried out. Dietary diversity was found to be low in 17.5% of the households, medium in 61.2%, and high in 21.3%. The household head’s age, education, income, home gardening skills, access to irrigation, and awareness of dietary diversity all had a positive and significant impact on household dietary diversity, but distance from the market had a negative impact. In addition, [29] analyzed household dietary diversity and its determinants in Finote Selam town, north west Ethiopia. Cross-sectional data was gathered from 400 households between 22nd to 30th August 2015. A logistic regression model was used to determine the factors related to household dietary diversity. Low, medium, and high dietary diversity scores were found in 11.8%, 67.2%, and 21% of the households, respectively. Family head (male-headed), eating frequency, and lack of cooking water were found to be substantially correlated with dietary diversity in households.

Ref. [30] examined 479 smallholder farmers in the Zimbabwean provinces of Manicaland and Masvingo’s level of crop diversification as well as the factors that contribute to it. Estimating diversity was done using the Herfindahl index, and factors related to agricultural diversification were assessed using the Tobit model. According to the gender results, households headed by men were somewhat more diverse than households headed by women. According to the Tobit model, agro-ecological zone, household income, farmer-to-farm extension, routine extension, farming experience, access to markets, membership in a farmers group, gender of the head of the household, education, number of livestock units, access to irrigation, and farms on level terrain are all important factors in increasing crop diversification.

Lastly, [18] examined factors affecting dietary diversity in the Usambara Mountains, Tanzania. The authors employed in-depth interviews, focus group discussions, and participant observation. Researchers found that participants strongly agreed on the importance of dietary diversity in maintaining and enhancing hunger over days, months, and seasons. According to locals, having enough financial resources, agrobiodiversity, terrain heterogeneity, and a diversity of livelihoods all helped them be able to eat a diversified diet and maintain decent nutritional status. Seasonality (climate change), household size, tradition/cultural beliefs, and gender were other factors that influenced nutrition and dietary diversity.

### Conceptual framework

**Fig. 1:**
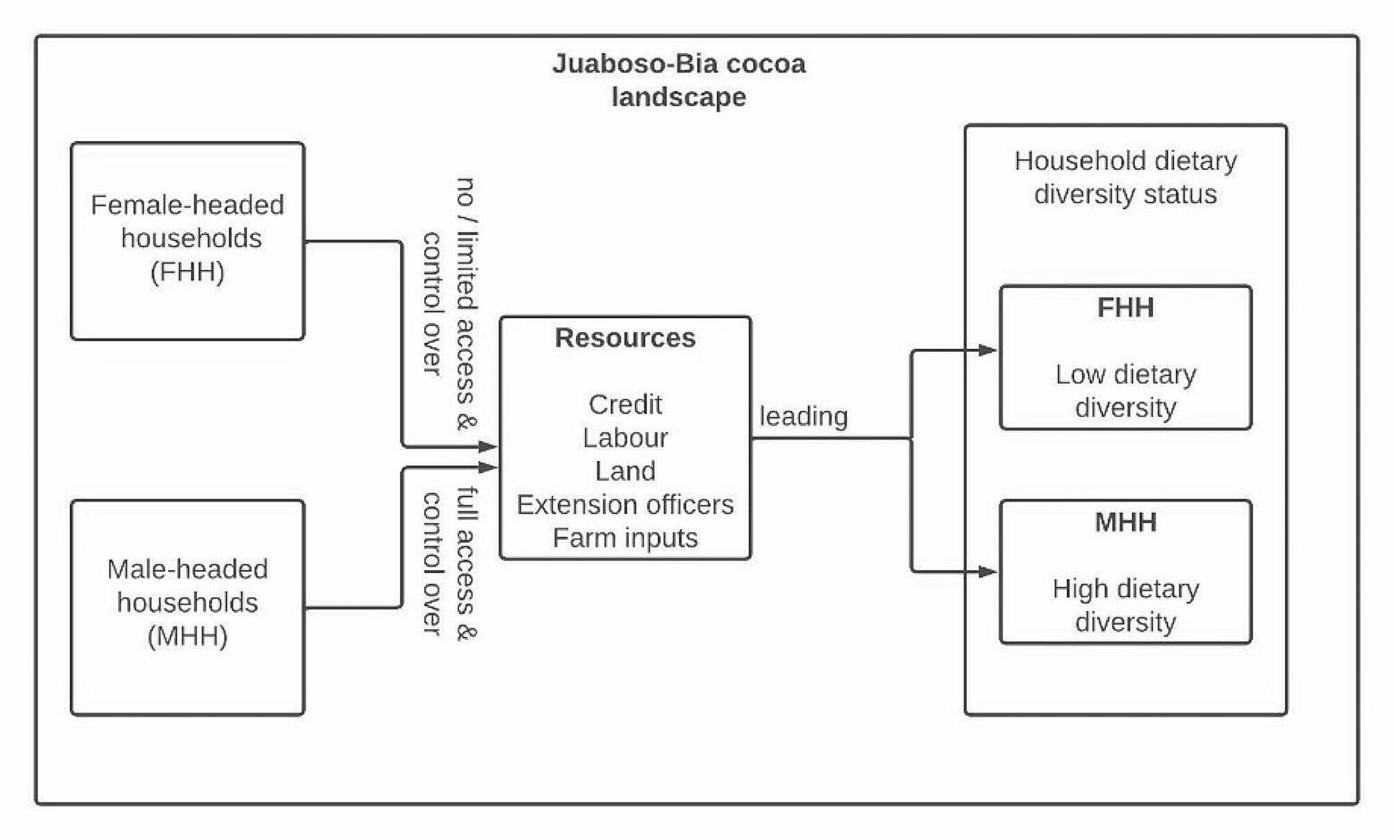
Conceptual framework illustrating factors influencing food security and dietary diversity status, with a focus on differences between female and male-headed households. *Source*: (adapted 31])

This study employed the conceptual framework by [31] to explain the relationship between gender and food security. Food and nutrition security status of female and male-headed households are diverse globally, nationally, and locally [31–33]. The differences in the food or nutritional security status of male and female-headed households are influenced by access to and control over resources or assets, such as land, agricultural inputs, fiancé, and labor [1, 31]. This situation is no different from that of the Juaboso-Bia cocoa landscape (Fig. 1). Female-headed households may have limited or no access to resources, such as credit, labor, land, extension officers, and farm inputs, which in the long run may lead to low dietary diversity and food insecurity [27]. The male-headed households may have full access to and control over resources and that will improve and cause those households to have high dietary diversity and be food secure.

## Methods

### Location of the study area

**Fig. 2:**
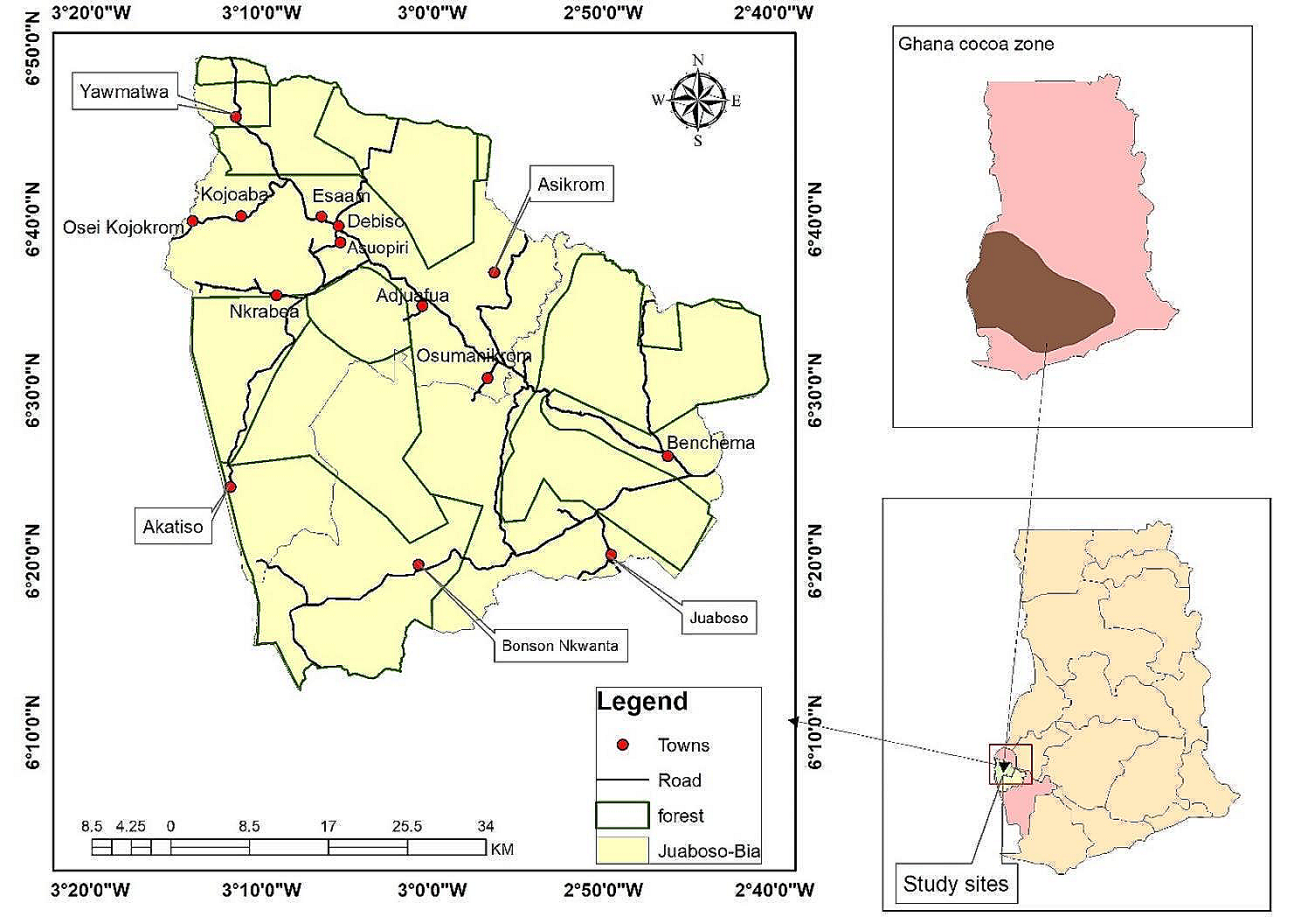
Map showing the Juaboso-Bia cocoa landscape within the Western Region of Ghana

The Juaboso-Bia cocoa landscape is one of the cocoa landscapes in the Westward of Ghana. The Westward contributed approximately 43% of the total 766,977 tons of annual regional cocoa purchases in 2019/2020 to Ghana’s cocoa production [34]. The Juaboso-Bia cocoa landscape encompasses Bia East, Bia West, and Juaboso districts, but for this study, only Bia West and Juaboso districts were studied. The studied area is located in the Western North Region of Ghana. The area lies within 2° 40’ W and 3° 20’ W longitude, and 6° 10’ N and 6° 50’ N latitude (Fig. 2). The studied landscape is endowed with natural resources, such as the Bia National Park and the Krokosua Hills forest reserves [35]. The population of the landscape is estimated to be 147, 374 by [35]. The major rainfall season is between May and June and the minor season is from September to October [8]. The occupation of the inhabitants is predominately agriculture, with cocoa being the largest cash crop cultivated by almost every household [35]. The other food crops grown are plantain, cassava, yam, cocoyam, oil palm, maize, rice, etc. Some households occasionally intercrop these food crops with cocoa farms, while others produce them on a small parcel of land for livelihood. The Juaboso-Bia is regarded as one of the hotspot zones for cocoa expansion, which is affecting food crop production [8–10, 36]; thus was selected to study the food security and dietary diversity status of male and female-headed cocoa household heads.

### Research design

Mixed research methods integrate qualitative and quantitative research designs employed to analyze phenomena and may occur in different approaches [37]. With this technique, it is possible to assess the consistency of data collected using a variety of instruments, compare and contrast one method’s findings with another, and show how the results of one approach can affect other research strategies, for example, qualitative data could help researchers to well describe their findings from quantitative data [37, 38]. The mixed methods ensure a comprehensive understanding and enhance the validity and credibility of the research [37]. For the mixed methods, the quantitative aspect through the use of questionnaires was used to find answers to the questions, namely household head dietary diversity and food security status distribution, sources of food, food production status, factors affecting crop diversification, and food shortage periods. The qualitative approach (in-depth interviews) was used to find answers to these questions, such as reasons/themes for low household dietary diversity/food security status, opinion on crop diversification, and their food unavailability experiences and production status. This qualitative approach was necessary since it captures the experiences and views of respondents.

### Data collection

This study collected primary data from the cocoa farmers in the Juaboso-Bia landscape in the form of quantitative and qualitative data. With the quantitative method, the sample size was calculated using the formula by [39], that is **n = (N / 1 + N(a)**^**2**^**)** because this formula ensures that the sample size is sufficient to provide reliable and accurate results. Where the margin error (**a**) was 5%, the sample size (**n**), and the sample population (**N**) was 15,491 cocoa households. The confidence level was 95% since this study involved human beings whose truthfulness of the information is vulnerable to prejudices. Now, sample size = (15,491 / 1 + 15,491 (0.05)^2^) = 390 households. The study interviewed 200 out of the 390 households due to largely limited financial resources and road inaccessibility. The study administered 200 semi-structured questionnaires in the Juaboso-Bia landscape. Following [1, 40–42], and [43], the multi-stage sampling technique was used to select the cocoa household heads from these communities, particularly, Yawmatwa, Adjuafoa, Kojoaba, Asuopiri, Akaatiso, Nkrabea, Bechema, Bonso Nkwanta, and Juaboso. With the multi-sampling technique, firstly, the study area was divided into 4 groups namely north, south, east, and west to ensure geographical representation and reduce biases. Secondly, purposive sampling was used to select the aforementioned communities based on road accessibility. Lastly, the simple random technique was applied to select the cocoa household heads to be interviewed. The semi-structured questionnaires (see supplementary material 1) covered the household heads’ dietary diversity score, food shortage periods, food production status, and demographic data (sex, age, education, etc.).

With the qualitative method, an in-depth interview data collection method was utilized, for instance, the usage of an in-depth interview approach aids in understanding the realities and social worlds that actors encounter daily, including their lived experiences, complexities, negotiations, views, conflicts, and shared meanings [44], as well as give way to the power dynamics, desires, and motives that shape how certain places, people, and events are portrayed [38]. The purposive sampling technique was used to select 14 participants to engage in the in-depth interviews, which comprised 7 male and 7 female-headed cocoa households that had over 20 years of cocoa farming experience. The study selected 2 participants (a male and female) from each of these purposively selected communities namely; Juaboso, Bonso Nkwanta, Benchema, Adjuafua, Yawmatwa, Essam-Debiso, and kojoaba.

### Semi-structured questionnaires analysis

The survey data was numbered, cleaned, and coded in Statistical Package for Social Sciences (SPSS) for the following analysis.

#### Dietary diversity and food security status analysis

The food security and dietary diversity status of the cocoa household heads were measured using the Household Dietary Diversity Score (HDDS) [17, 21, 45]. The HDDS included 12 local food groups (see Appendix 1 ) that were used to analyze the dietary diversity and food security of the male and female-headed cocoa households in the study area. A household was scored 1 if that household consumed any of the 12 food groups in the past 24- hours, otherwise 0. The total HDDS was 12, which was further categorized into low dietary diversity (0–4 food groups); medium dietary diversity (5–8 food groups); and high dietary diversity (9–12 food groups). For the food security status estimation, any household that scored less than 6 HDDS was classified as food insecure, while more than 6 HDDS was classified as food secure [16, 21]. Also, other concepts of food security, such as food availability and accessibility were analyzed. The results were presented in descriptive statistics. Chi-Square was used to test the association between food security status and household head gender.

#### Variables affecting crop diversification

A binary logistic regression model was utilized to predict the surveyed household heads’ socio-economic factors influencing their opinions on the impact of cocoa expansion on crop diversification in the study area, following several studies [8, 21, 25]. The binary logistic regression model was CECD_i_ = β_i_ + β_1_ × _1_ + β_2_ × _2_ + β_3_ × _3_ + β_4_ × _4_ + β_5_ × _5_ + β_6_ × _6_ + β_7_ × _7_ + β_8_ × _8_ + β_9_ × _9_ + β_10_ × _10+_e….(1) where; CECD = Cocoa expansion affects crop diversification (No “0”, Yes “1”); β_i_ = the constant term; β_1−10_ = the coefficient of the independent variables. The independent variables used in the model included:

X_1_ = Sex of the household head ( Female “0”, Male “1”).

X_2_ = Age of the household head in years.

X_3_ = Education status of the household head (No formal education “0”, Formal education “1”).

X_4_ = Marital status of the household head ( Other “0”, Married “1”).

X_5_ = Household size in numbers.

X_6_ = Cocoa cooperation membership ( No “0”, Yes “1”).

X_7_ = Cropland conversion (No “0”, Yes “1”).

X_8_ = Credit access (No “0”, Yes “1”).

X_9_ = Total household income in cedis.

X_10_ = Access to extension officers (No “0”, Yes “1”).

### Respondents’ in-depth interviews analysis

The information from the interviews was categorized into themes and used to validate and support the quantitative findings. The thematic analysis was employed to identify the factors influencing low dietary diversity and food insecurity among male and female-headed cocoa households.

## Results

### Respondent’s socio-economic characteristics

The responses (Table 1) from the surveyed household heads showed that 39.5% and 60.5% were headed by females and males, respectively. Most of the interviewed household heads aged between 46 and 66 years (58.5%). For the educational levels of the household heads, 10%, 26.5%, 13.5%, and 17.5% obtained primary, JHS/Form 4, SHS/SSS, and Tertiary, respectively, as well as 32.5% had no formal education. Moreover, most of the respondents had access to extension officers (87%) and credit (70.5%). Also, 81.5% of the household heads had converted their cropland to cocoa farms, and 75.5% of them had joined cocoa cooperative groups. In terms of the total household income, the majority of the surveyed household heads earned less than 10,000 cedis (52.5%). Furthermore, 90.5% of the household heads perceived that cocoa expansion affects crop diversification in the study area.

**Table 1:** Socio-economic characteristics of the household heads in the Juaboso-Bia cocoa landscape

Socio-economic variables		Valid frequency	Valid percent (%)	Mean
Sex	Female	79	39.5	0.61
	Male	121	60.5	
Age	between 25 and 45	44	22	56.14
	between 46 and 66	117	58.5	
	above 67	39	19.5	
Education	Primary	20	10	0.68
	JHS/Form 4	53	26.5	
	SSS/O or A-level	27	13.5	
	Tertiary	35	17.5	
	No formal education	65	32.5	
Marital status	Married	148	74	0.76
	Single	8	4	
	Divorced	14	7	
	Widow/widower	30	15	
Household size	between 1 and 5	55	27.5	8.14
	between 6 and 10	98	49	
	above 10	47	23.5	
Cocoa cooperation membership	No	43	21.5	0.79
	Yes	157	78.5	
Cropland conversion to cocoa	No	37	18.5	0.81
	Yes	163	81.5	
Credit access	No	59	29.5	0.70
	Yes	141	70.5	
Access to extension officers	No	26	13	0.87
	Yes	174	87	
Total household income	less than 10,000	105	52.5	1.82
	Between 10,000 and 30,000	26	13	
	Above 30,000	69	34.5	
Household heads’ perception				
Cocoa expansion affects crop diversification	No	19	9.5	0.90
	Yes	181	90.5	

Source: Fieldwork, 2023

### Distribution of dietary diversity and food security pattern among cocoa household heads in the Juaboso-Bia cocoa landscape

**Fig. 3:**
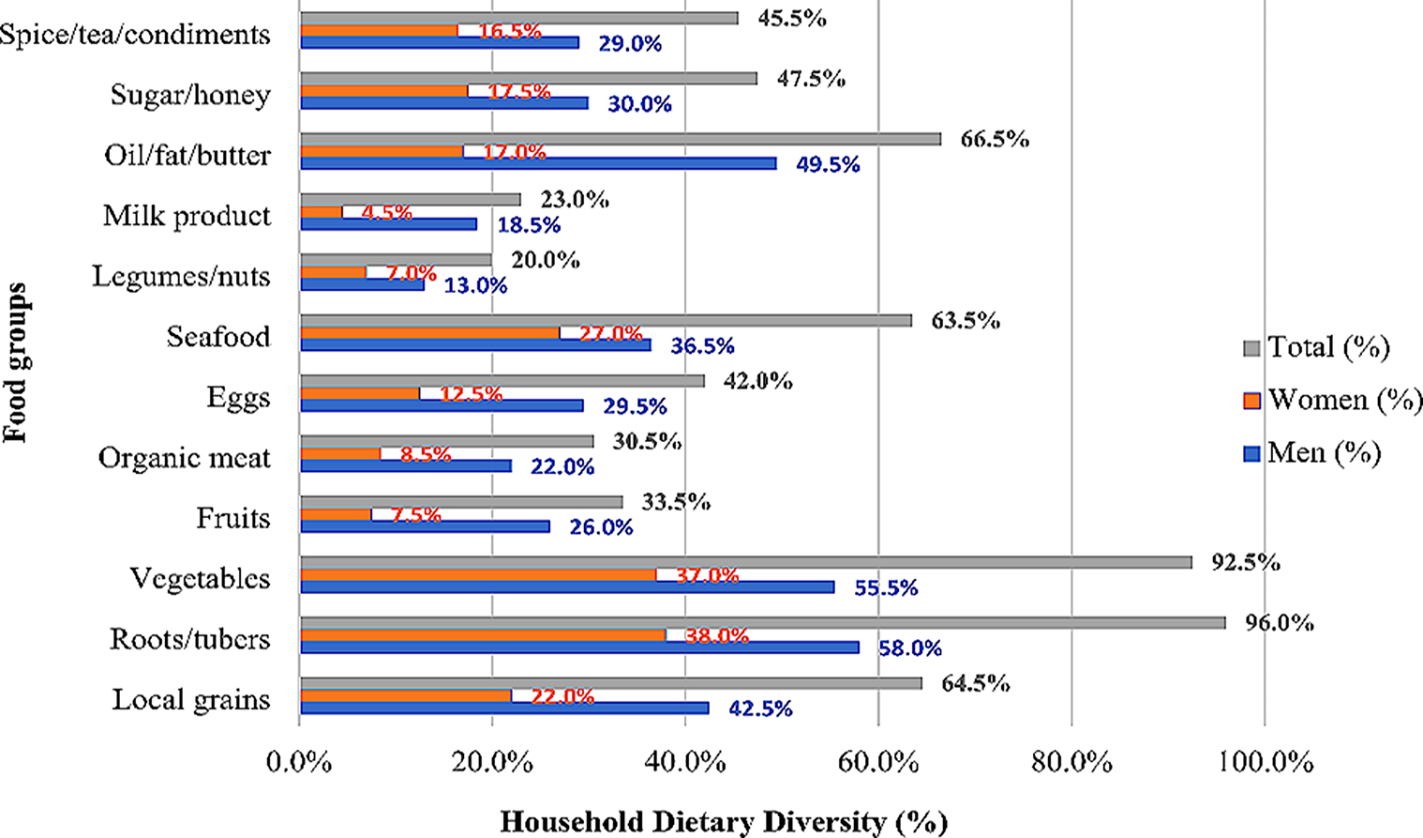
Recalled household food groups eaten in male and female-headed cocoa households. *Source*: Fieldwork, 2023

In the Juaboso-Bia cocoa landscape, it was observed that there was a disparity distribution in the amount of the 12 food groups consumed in households headed by women than that households headed by men. According to Fig. 3, the male-headed households recalled eating roots/tubers (58%), vegetables (55.5%), oil/fat/butter (49.5%), local grains (42.5%), seafood (36.5%), and sugar/honey (30%), respectively. These food groups were averagely consumed in male-headed households, particularly organic meat, fruits, spice/tea/condiments, and eggs, ranging from 22 to 29.5%. Legumes/nuts (13%) and milk products (18.5%) were consumed less. In an interview with a participant, it was indicated that;

*“We eat vegetable stew with cassava, yam, cocoyam, and maize most of the time because it is our basic diet. I grow these foods on my family farm, so I don’t always have to buy them. I get these food items from the market when I run out because they are inexpensive”(Key informant interview, 23 January 2023).*

The households headed by women also reported these food groups being consumed the most namely roots/tubers (38%) and vegetables (37%). The food groups consumed on average were seafood (27%), and local grains (22%). The least consumed food groups were sugar/honey (17.5%), oil/fat/butter (17%), spices/tea/condiments (16.5%), eggs (12.5%), organic meat (8.5%), fruits (7.5%), legumes/nuts (7%), and milk product (4.5%). For example, a female headed-household noted that;

*“I never really consider the meats or eggs (quality) in the meals I offer to my kids because I’m always more concerned about the quantity. We can buy a lot of cassava or maize and that will provide us with enough food to last us for approximately a month with the money that will be used to purchase meats, milk, eggs, fruits, and legumes. These food groups are extremely expensive to buy here on the market, and they won’t even satisfy my kids as much as local cereals and roots/tubers do”(Key informant interview, 20 January 2023).*

According to Table 2, low dietary diversity was more prevalent in female-headed households (60.8%) than the male-headed households (37.2%). Medium and high dietary diversity categories were high in the male-headed households, specifically 30.6% and 32.2%, compared to the female-headed households, such as 19% and 20.2%, respectively.

**Table 2:** Dietary diversity status among the male and female-headed cocoa households in the Juaboso-Bia cocoa landscape

Household dietary diversity status	Gender	
	Female	Male
Low dietary diversity (0–4)	60.8%	37.2%
Medium dietary diversity (5–8)	19%	30.6%
High dietary diversity (9–12)	20.2%	32.2%
Total	**100%**	**100%**

Source: Fieldwork, 2023

Following Table 3, 29.1% of the female-headed cocoa households were food secure as compared to food insecure (70.9%). Conversely, 52.1% of the male-headed households were food insecure while 47.9% were food secure. With the chi-square test, the gender group comprised male and female-headed households, which was compared to food security status that is secure and insecure. The p-value for the chi-square analysis was statistically significant, therefore the null hypothesis was rejected. There is a significant relationship or difference between food security status and the gender of the household head.

**Table 3:** A Chi-Square test statistics on the relationship between food security status and gender of the household head

Food security status and gender cross tabulation					
Food security status			**Gender**		
			**Male**		**Female**
Secure			47.9%		29.1%
Insecure			52.1%		70.9%
Total			**100%**		**100%**
**Chi-Square Tests Statistics**					
	**Value**	**df**	**Asymp. Sig. (2-sided)**	**Exact Sig. (2-sided)**	**Exact Sig. (1-sided)**
Pearson Chi-Square	9.575^a^	1	0.002		
Continuity Correction^b^	8.691	1	0.003		
Likelihood Ratio	9.774	1	0.002		
Fisher’s Exact Test				0.002	0.001
Linear-by-Linear Association	9.527	1	0.002		
Number of Valid Cases	200				

### Food unavailability experience of male and female-headed cocoa households

Some of the male-headed households (66.1%) and female-headed households (83.5%) indicated that they face food shortages or unavailability in the study areas (Table 4). They expressed that food shortage or unavailability experience could be severe, average, and low according to certain months.

**Table 4:** Food unavailability experience of male and female-headed cocoa households in the Juaboso-Bia cocoa landscape in 2023

Food unavailability experience	Gender	
	Female	Male
Yes	83.5%	66.1%
No	16.5%	33.9%
Total	**100%**	**100%**

Source: Fieldwork, 2023

**Fig. 4:**
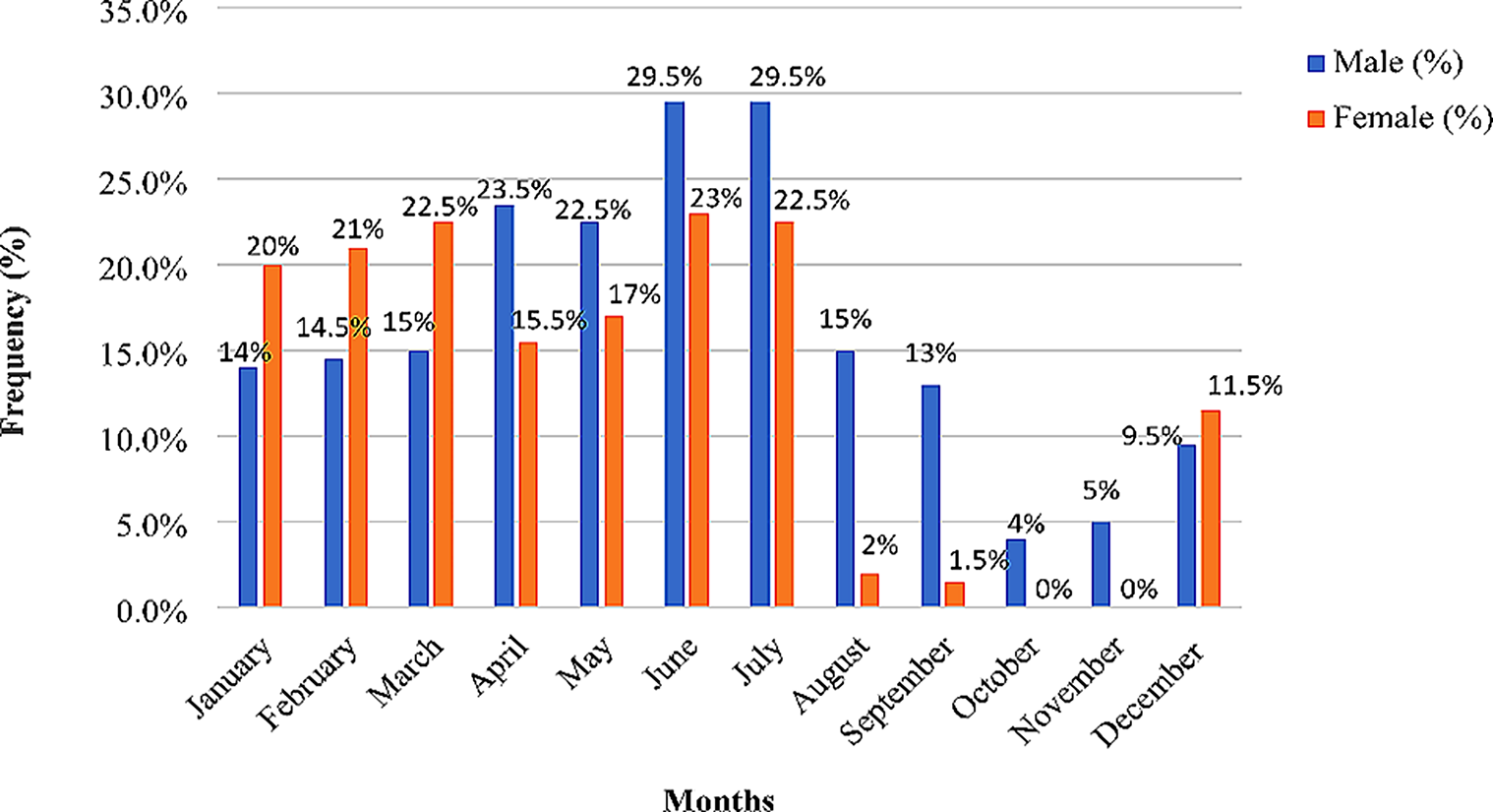
Food unavailability months per gender of the household head in the Juaboso-Bia landscape. *Source*: Fieldwork, 2023

The months that the male-headed households recalled facing severe food unavailability were July, June, April, and May, however, for the female-headed households, the severe months were June, July, March, February, and January (Fig. 4). The average severe food shortage months were March, August, February, January, and September for the male-headed households, and May, April, and December for female-headed households. The low severe food unavailability months for female-headed households were August, September, October, and November. On the other hand, December, November, and October were less recorded food shortage months for households headed by men. For instance, in an interview with the head of a cocoa household, he shared that;

*“When the rainy season approaches, especially in June and July, I am really frightened because the rains are exceptionally heavy and threaten to destroy my food crops. Also, the food I store for the rainy season is consumed quickly, causing my family to eat fewer meals each day. We occasionally go without food to eat. My home experiences food shortages throughout the dry season in December to March because the weather is so dry that the crops are unable to flourish” (Key informant interview, 20 January 2023)*.

### Sources of staple food, accessibility to food, food production status, and dietary diversity nexus among cocoa household heads

**Fig. 5:**
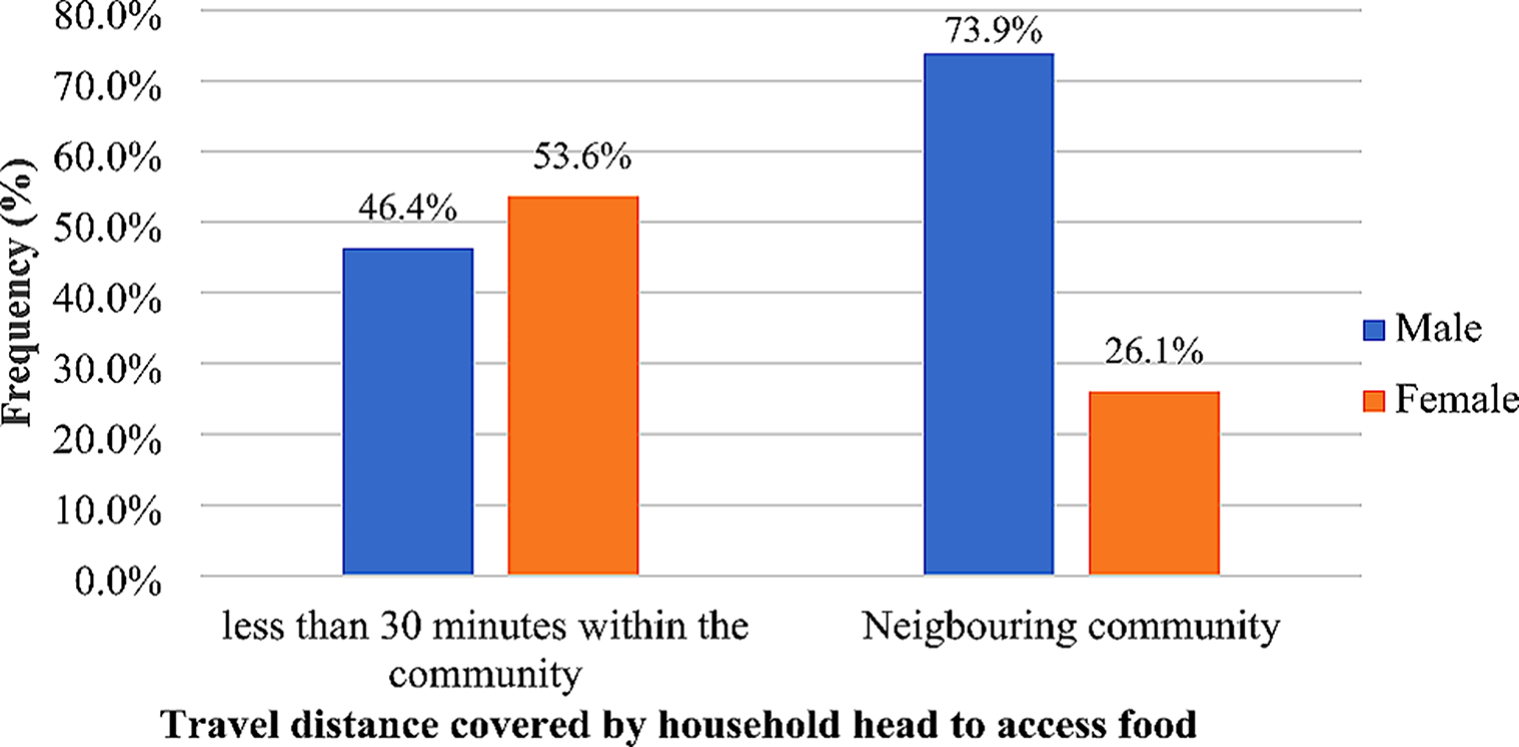
Travel distance covered by the sex of the household head to get staple food. *Source*: Fieldwork, 2023

Households headed by males and females in the studied area expressed that they obtain their staple foods through self-production, market, both self-production and market and others. According to Tables 5 and 81% of the female-headed households got their food from both self-production and the market, 10.1% from self-production, 8.9% from the market, and no household indicated through others (0%). In addition, for the female-headed households that said they obtained their food from the market, 53.6% of the households walked less than 30 min within the community and 26.1% got it from the neigbouring communities (Fig. 5).

**Table 5:** Sources of staple food for male and female-headed households

Sources of staple food	Gender	
	Female	Male
Self-production	10.1%	30.6%
Market	8.9%	4.1%
Self-production and market	81.0%	64.5%
Others	0%	0.8%
Total	**100%**	**100%**

Source: Fieldwork, 2023

Interestingly, for the male-headed households, 0.8% of them obtained food through others, while 4.1%, 30.6%, and 64.5% had food via market, self-production, and both market and self-production, respectively. Also, out of the male-headed households that stated they purchased their food at the market, 46.4% claimed to have walked less than 30 min within the community to get food and 73.9% got their food from neigbouring communities (Fig. 5).

According to Table 6, within the households that obtained their foodstuffs solely from the self-production source, 34.4% had low, 19.3% had medium, and 5.5% obtained high dietary diversity categories. For households that relied on the market for staple food, 10.8%, 3.8%, and 0% encountered low, medium, and high dietary diversity categories, respectively. Also, 53.8%, 76.9%, and 94.5% experienced low, medium, and high dietary diversity categories, respectively in households that depended on both the market and self-production for foodstuffs.

**Table 6:** Sources of local food and dietary diversity status nexus

Sources of staple food	Dietary diversity status		
	Low	Medium	High
Self-production	34.4%	19.3%	5.5%
Market	10.8%	3.8%	0%
Self-production and Market	53.8%	76.9%	94.5%
Others	1.0%	0%	0%
Total	**100%**	**100%**	**100%**

Source: Fieldwork, 2023

A respondent shared that;

*“Hahaha! I cannot cultivate all the food crops in this whole place. So, I grow some staple crops, such as cassava, plantains, cocoyam, tomatoes, garden eggs, and pepper, and buy protein, condiments, and fruits like chicken, meat, milk, fish, and oranges from the market. Thus, supplementing my self-production with goods from the market boosts the quality of diet my household and I consume” (Key informant interview, 26 January 2023).*

**Fig. 6:**
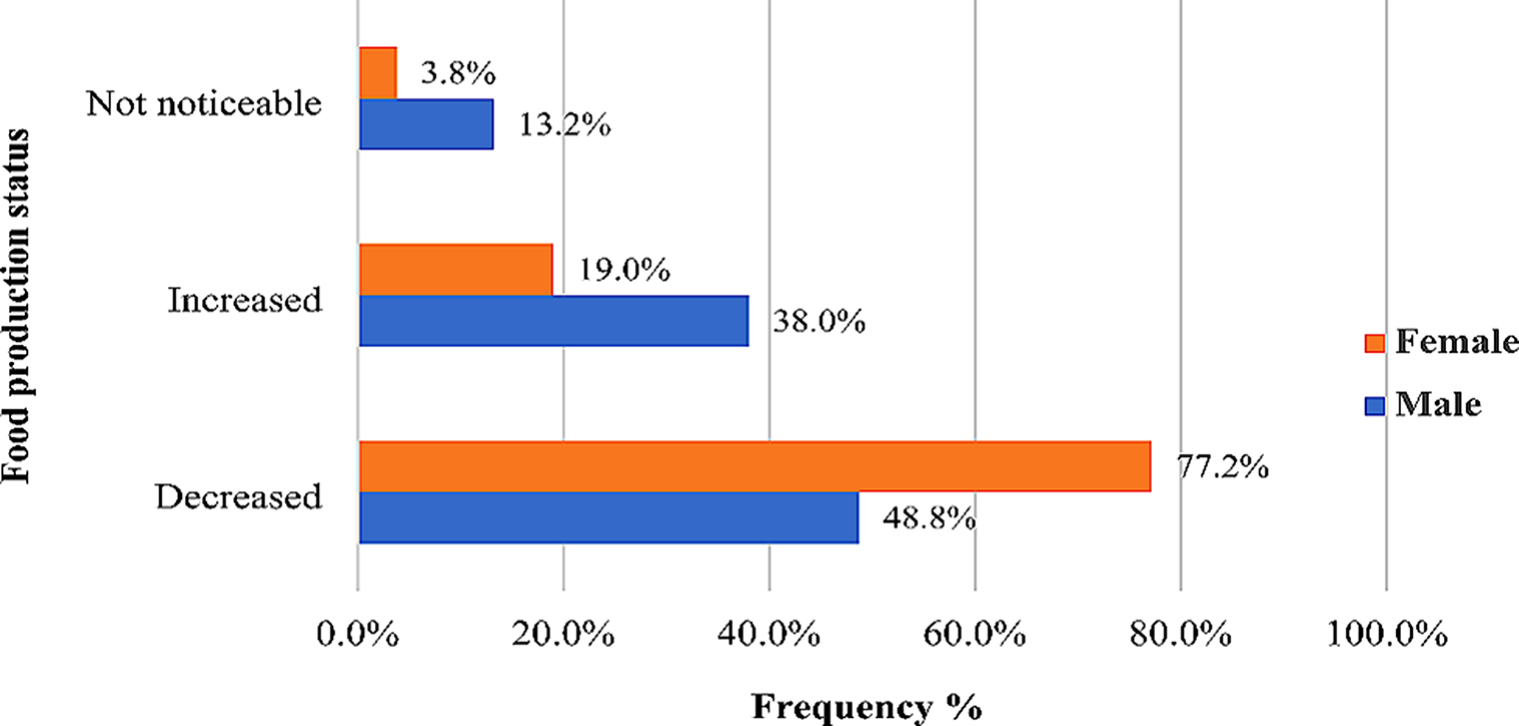
Food production status for the past 5 years among male and female-headed cocoa households. *Source*: Fieldwork, 2023

Concerning the food production status for the past 5 years (Fig. 6), the study revealed that the female-headed households had 3.8%, 19%, and 77.2% as not noticeable, increased, and decreased, respectively. The male-headed households, on the other hand, had 48.8% as decreased, 13.2% as not noticeable, and 38% as increased.

With regards to Table 7 results, the households that experienced “not noticeable” food production status had 5.4%, 25%, and 1.8% as low, medium, and high dietary diversity, respectively. Again, households with decreased food production status witnessed 94.6% as low, 55.8% as medium, and 5.5% as high dietary diversity status. The majority of the households that obtained increased food production status had 92.7% high dietary diversity status, with only 0% and 19.2% as low and medium dietary diversity status, respectively.

**Table 7:** Food production status and dietary diversity status nexus

Food production status	Dietary diversity status		
	Low	Medium	High
Not noticeable	5.4%	25%	1.8%
Decreased	94.6%	55.8%	5.5%
Increased	0%	19.2%	92.7%
Total	**100%**	**100%**	**100%**

Source: Fieldwork, 2023

In an interview with a household head who had increased food production status disclosed that;

*“Over the past 5 years, my household members and I have managed our piece of land very well by practicing sustainable farming methods. We managed to grow cocoa and a variety of food crops on the small land. Because of that, my household eats different diets daily and has enough food always, which has ensured my household’s food and nutrition security” (Key informant interview, 2 February 2023).*

### Multifaceted themes influencing low dietary diversity and food insecurity in male and female-headed cocoa households in the Juaboso-Bia cocoa landscape

#### Climate change/ variability

Climate change/ variability was among the common themes that emerged from the interviews, which has negatively impacted many of the cocoa households. For instance, through this study, it was revealed that the food diversity of both male and female-headed households is impacted by some seasonal variations that do not make certain crop types flourish. One of the interviewees stated that;

*“I remember when I was young, my father used to cultivate a lot of foodstuffs even to the point of selling some to other households. This was because there was an abundant and reliable rainfall pattern. However, in this era, the rains fall unexpectedly and heavily to the extent of destroying most grown crops on my farms. Due to this, my household eats what is available and mostly not rich in vitamins and not a balanced diet” (Key informant interview, 20 January 2023).*

Another household head mentioned that;

*“Climate change has resulted in the death of our cocoa trees and food crops since the crops receive too much sunlight hence declining the yields generated from cocoa and food production. Due to the sudden arrival of the rains and dryness, we are currently unsure of what to plant. Even some of our poultries die as a result of excessive heat and do not get to eat their products” (Key informant interview, 22 January 2023).*

#### Poverty

The results of the study showed that many of the cocoa farmers expressed that they lacked the money to diversify their diets and also to buy farm equipment to boost their food production in the study area. In the course of the interviews, it was disclosed that financial constraints limited the variety of food the household heads provided to their members. For example, these two participants respectively stipulated that:

“*Hmm!, I have been farming cocoa for almost 3 decades and I cannot boast of eating a well-balanced diet every day. My household only eats starchy foods, such as cassava, plantain, yam, and vegetables, such as tomatoes and garden eggs, because that is what I can afford”* (*Key informant interview, 26 January 2023)*.

*“Sometimes, I feel shy to tell my non-cocoa farmers friends that I have not even eaten chocolate before, and I consume chocolate drinks occasionally. I cannot even feed my household members with cheap food, for example, “abom” (literally means vegetable stew with either yam or cocoyam or cassava) let alone talk of eating fruits, eggs, and meat. Due to traders’ perception that this area is a wealthy cocoa district, these food items are very expensive to purchase. Also, buying modern farm tools, fertilizers, and hiring labor is expensive, which affects our food crop and cocoa production ”* (*Key informant interview, 2 February 2023)*.

#### Unavailable lands

Lack of land was also a key dimensions that was revealed from the interviews with the participants. The study found that the unavailability of land has hindered many household heads from growing a variety of crops, hence has influenced the diets consumed by these households. The interviewees noted that the Juaboso-Bia landscape is full of forests and cocoa plantations, and they cannot either go into the forest to cultivate food crops or cut down their cocoa trees to grow food crops. For example, in an interview with a respondent, it was revealed that;

*“Currently, we have used all the lands for cocoa production and there is no available land in this district to grow food. I gave up my land for cocoa production to increase the income and food diversity for my family, but that goal was never realized, and now my family is starving and unable to consume cocoa because it is not edible” (Key informant interview, 1 February 2023).*

In addition, one participant said that;

*“The government through the forestry commission allocated portions of the off-reserved forest to us to cultivate our food crops some time back. The condition was to replant trees after harvest before shifting to a new place to begin another cultivation. Failure to do so will lead to the prevention of cocoa farmers from entering the off-reserved to grow food crops. Some farmers failed to comply with the condition regardless of the number of warnings given, therefore we were restricted from growing food crops there and this has affected my household food production” (Key informant interview, 26 January 2023).*

#### Large household size

According to the findings of this study, large household size was among the themes that promoted low dietary diversity in the Juaboso-Bia landscape. The respondents expressed that household heads with large family sizes find it cumbersome to provide quality and different food groups for their households compared to household heads with small household sizes. For instance, during the interview, one participant indicated that;

*“I have about 10 household members and due to that I barely buy food items rich in vitamins because those food items do not satisfy me and my family. Thus, I regularly buy less expensive food usually starchy food, which is not well rich in vitamins since all my children can eat and get satisfied” (Key informant interview, 31 January 2023).*

#### Gender stereotypes/ beliefs

One of the key facets that played a major role in promoting low dietary diversity among female and male-headed households was gender stereotypes/ beliefs, according to the study’s findings. The interviews revealed that both men and women face gender stereotypes and that has prevented many of them from working hard to provide good diets for their household members.

One participant shared that;

*“Before the demise of my husband, we ate a well-balanced diet almost every day. After his death, the household responsibility has become very heavy for me to feed myself and my children balanced diets. Because I am the head of the family, I am most times denied access to some of the cocoa farms, extension officers, and other resources to increase my cocoa yields, hence boosting my household dietary diversity” (Key informant interview, 24 January 2023).*

In another interview with one of the participants, he revealed that;

*“I do occasionally find it challenging to give my family a variety of foods to consume every day to grow well and strong, even though I am a man and the leader of my household. This is because some of my male friends point hands at me and call me all sorts of names for having a small land size, being unable to cultivate different crops, and being a cocoa care-taker for other households headed by men” (Key informant interview, 28 January 2023).*

### Perceptions of the cocoa household heads on the implication of cocoa expansion on crop diversification in the Juaboso-Bia cocoa landscape

Table 8 below provides the parameter estimates for the binary logistic model. Among the 10 variables used to estimate the logistic model, only 3 variables were statistically significant, namely sex, cropland conversion to cocoa, and total household income.

**Table 8:** Logistic regression model predicting the influence of household heads’ socio-economic characteristics on their perceptions of the impact of cocoa expansion on crop diversification in the Juaboso-Bia landscape

Variables	Coefficient	Significance	Odd ratio
Constant	4.691	0.019	108.938
Sex (Male)	-0.982	0.10*	0.405
Age	-0.041	0.196	0.960
Education status	-0.338	0.562	0.713
Marital status	-1.215	0.164	0.279
Household size	0.096	0.718	1.101
Cocoa cooperation membership	-0.552	0.452	0.576
Cropland conversion	1.079	0.093*	2.942
Credit access	0.681	0.206	1.975
Total household income	0.645	0.050**	1.906
Access to extension officers	-1.290	0.263	0.275
Pseudo R2	0.71		
-2 Log Likelihood	85.46		
Significance	0.007		
Observation	200		

Significance * (10%), **(5%), ***(1%)

In line with this, a respondent transpired that;

“*The cocoa has eaten our croplands, so I do not have a variety of food to eat. The land I could have used to grow crops, such as cassava, vegetables, yam, and rice, I have used it to expand my cocoa farm. I believe when we continue to expand cocoa, we will one day wake up with no different kinds of food to consume. Because of this, the prices of foodstuffs on the market are expensive to buy” (Key informant interview, 28 January 2023).*

Another household head indicated that;

*“A few of the cocoa households here do crop diversification. The reason why this has not been achieved is that the cocoa sector is lucrative that is we earn more than cultivating only food crops. I remember when I used to do more food crop farming, I used to find it difficult to get people to come and buy the foodstuffs so most of them ended up spoiling, and that affected my income. Hmm, so I decided to do more cocoa farming, and I earn a lot from it. Regardless, this to a large extent is affecting the variety of diets my household eats” (Key informant interview, 31 January 2023).*

## Discussion

### Household head gender and its implication on dietary diversity distribution and food security pattern

The findings from this present study suggest that roots/tubers, vegetables, oil/fat/butter, local grains, seafood, and sugar/honey were highly consumed in male-headed households, whereas only roots/tubers and vegetables were mostly eaten in female-headed households. This food group pattern indicates that both male and female-headed households heavily rely on roots/tubers, local grains, and vegetables because of their low cost and self-production [2]. This finding was similar to that of [2] in Ecuador and [18] in Tanzania (East Usambara Mountain). However, it disagreed with the findings of [24], who revealed that green vegetables are hardly eaten in rural households.

The seafood group, such as fish which is rich in protein and is good for human growth and development was highly consumed in male-headed households and average in female-headed households. This could be partially explained by the fact that male-headed households had more income sources and financial capacity than female-headed households [31]. This present finding was inconsistent with the findings of [1] in Tanzania, [46] in Botswana, and [3] in Mexico. Also, [19] found that female-headed households consumed more seafood than male-headed households, which was contrary to this present study.

Food groups namely eggs, milk products, organic meat, legumes/nuts, and fruits are essential for the human body daily because of their protein, vitamins, and minerals content [47]. Regardless, these food groups were least eaten in female-headed households, which is largely due to lack of income or poverty, and was corroborated by the findings of [2]. The lack of this food group consumption in female-headed households could imply that such households experience malnutrition [15]. On the other hand, these food groups were relatively highly consumed in male-headed households, and this finding was contrary to that of [1].

Concerning the household dietary diversity status of this study (Table 2), the general dietary diversity pattern distribution was skewed towards the males and unfairly towards the female-headed households. Specifically, the low dietary diversity was more widespread in female-headed (60.8%) households than in their male counterparts (37.2%). The combination of medium and high dietary diversity categories in male-headed households was 62.8%, which is statistically greater than what female-headed households obtained (39.3%). Generally, the male-headed households had a relatively good dietary diversity (62.8%) even though 37.2% had low dietary diversity. This connotes that the male-headed households consumed relatively high dietary diversity, which improve their food and nutrition security than female-headed households. This evidence confirmed the findings of [24] and [1], who found that low dietary diversity is more frequent in female-headed households than in men-headed households in Uttar Pradesh State, India, and Tanzania (Bahi District and Mbarali District), respectively. In addition, this was not in agreement with the findings of [19], who revealed that female-headed households had relatively higher dietary diversity than male-headed households.

Moreover, findings from the study (Table 3) suggested that food insecurity is prevalent in female-headed cocoa households (70.9%) compared to their male counterparts (52.1%). This finding conforms to that of [21], who found that 61% of the female-headed cocoa households in Wassa Amenfi West District were more food insecure than male-headed households (33%). Also, the results in Table 3 confirmed the conceptual framework adapted from [31] in Fig. 1, which suggested that households headed by men are more food secure than female-headed households because male-headed households have control over resources, such as farm inputs, land, and labor. The findings also implied that cocoa households with food-secure status mostly have high dietary diversity and low dietary diversity is usually associated with households with food-insecure status.

### Food unavailability experiences in relation to gender of the household head

Food shortage or unavailability was prevalent in April, May, June, and July for male-headed households, but female-headed households experienced it in February, March, June, and July. Both households shared similar months of food unavailability, such as June and July. May, June, and July happen to be the major rainy season, which usually destroys food crops and make poor households vulnerable to food insecurity [8]. Again, due to the rainy months, from July to September, [27] discovered that households in the cocoa industry in Ghana go without food during those months, and the income generated by cocoa sales also runs out before the following harvest. The dry season which is from December to March dries most food crops and makes households food insure. Some households eat one meal, usually starchy or carbohydrate foods (such as cassava, yam, and cocoyam) a day during the long dry season. Similarly, [1] found that male-headed households in Tanzania’s Bahi District and Mbarali District had food shortages from mid-November to March, to the point that such households consume just one meal per day, typically consisting of ugali, cassava, and rice.

### Implications of sources of staple food on household dietary diversity status

The types or sources of food households rely on greatly determine the status of the dietary diversity of the household either low, medium, or high. As evident in Table 6, households that rely primarily on the market or self-production have a higher propensity to have a low level of dietary diversity because they may experience greater hardship during times of famine than households that depend on both self-production and the market. This implies that households need to supplement their self-production with produce from the market to ensure their food and nutrition security all year round. This finding is in line with that of [20], who revealed that while farm production diversity alone has a negligible impact on dietary diversity, it has a higher impact when combined with goods bought from the market.

### Exploring the multidimensional nature of low dietary diversity and food insecurity in male and female-headed cocoa households in the Juaboso-Bia cocoa landscape

#### Climate change/ variability

One of the key determinants of low dietary diversity identified by the study was climate change. The abrupt changes in the weather in the study area have negatively affected many cocoa household heads in diverse ways, leaving the afflicted households vulnerable to food insecurity and low dietary diversity. Seasonal variations usually do not make certain crop types flourish, which has impacted the food diversity of cocoa household heads. This finding was aligned with that of [18], who discovered that food scarcity or unavailability caused by changes in seasonality affects the diversity of the locals’ diets in the East Usambara Mountains, Tanzania.

#### Poverty

Both male and female-headed households agreed that poverty is an important factor that affects a household’s dietary diversity. A household with a higher income could afford to buy different crop seeds, buy more land, and hire more farm laborers on his or her farms, thereby increasing the dietary diversity and food security of the household. Many of the cocoa household heads expressed that they lacked the money to diversify their diets in the study area. Because of poverty, most of these households experienced low dietary diversity and food insecurity, and in other studies, similar results have been discovered [2, 8, 18].

#### Unavailable lands

Lands are a major resource needed for agricultural activities. A household’s dietary diversity can be influenced by the size of the land since a greater land area would allow for the growth of more different crops, protecting the household’s food security and dietary diversity [2]. The local people in the Juaboso-Bia landscape complained that there was no available land for them to cultivate food crops since they used all their lands for cocoa production. Most of them cultivate food crops under small cocoa trees, which is not enough for their sustenance, thus influencing their dietary diversity status. Similar findings were made by [8], who found that no land was available for crop farming in the Juaboso District because the cocoa farmers had turned their food cropland into cocoa farms for a variety of reasons that harmed their ability to feed themselves. Their studies further noted that some cocoa farmers grow food crops in portions under cocoa rehabilitation, and some households barely get food to eat in a day.

#### Large household size

The size of a household may increase or decrease the household head’s ability to provide more diverse food for his or her household members. The large household size of some of the male and female-headed cocoa households accounted for the low dietary diversity in the Juaboso-Bia landscape. Many studies have shown that large household size stands a higher chance of having food insecurity since the head of the household would have many mouths to feed [8, 18, 28, 29]. However, larger households may be better able to maintain a diverse diet since they will have access to more sources of income and hence more money to spend on different types of food [2].

#### Gender stereotypes/ beliefs

The study’s findings implied that gender beliefs had a significant impact on the food and nutrition security of the male and female-headed cocoa households in the Juaboso-Bia landscape. This finding supports the notion that both men and women in rural communities do face gender stereotypes or beliefs, which go a long way to affect their production, hence leading to low dietary diversity [1, 18]. For example, [1] disclosed that men in Tanzania also experience gender-based stereotypes, such as, the local people considered that men who take food home, namely vegetables or meat are controlled by their wives. Moreover, [18] asserted that although women who are household heads go through gender-based obstacles, many of them have broken the barriers to provide good diets for their households.

### Household heads’ socio-economic variables influencing their perception of the impact of cocoa expansion on crop diversification in the Juaboso-Bia cocoa landscape

According to Table 8, sex was 10% statistically significant in influencing the perception of the surveyed household heads’ of the effect of cocoa expansion on crop diversification. Sex had a negative relationship with the dependent variable. The result implies that for every rise in the number of households headed by females, the odd ratio decreases by a factor of 0.405, influencing those households to perceive that cocoa expansion does not affect crop diversification. This can be partially explained by the fact that in rural cocoa areas, households headed by women usually experience financial constraints and many responsibilities; hence they are motivated to expand their cocoa farms to reduce their burden in meeting their needs. This finding is supported by [30] who found that female-headed households are negatively associated with crop diversification, that is they do not grow different types of crops as compared to male-headed households in Zimbabwe.

Cropland conversion to cocoa had a statistical significance with the dependent variable at 5%. This result suggests that an increase in the rate of cropland conversion to cocoa would have a positive influence on the perception of household heads on the implication of cocoa expansion on crop diversification. That is, the odd ratio increases by a factor of 2.942; therefore influencing the household heads to perceive that cocoa expansion affects crop diversification. Cropland conversion to cocoa displaces croplands, limiting the ability of household heads to grow other types of food crops. As a result, this affects household food security and diet quality of the cocoa household heads. The study by [48] disclosed that when cocoa household heads increase the size of their cocoa farms, they are not able to grow different crops and, hence become more dependent on the markets for agricultural products, which raises the cost of food and jeopardizes their ability to feed their families. Also, this study’s finding was similar to that of [8], who revealed that about 80% of the cocoa farmers in the Juaboso District had converted their croplands into cocoa farms, which affected the variety of food they consume.

Total household income was positively related to the dependent variable at 10%. The finding implies that when the unit of household income increases, the odd ratio increases by a factor of 1.906; thus influencing the perception of those household heads that the expansion of cocoa farms hinders crop diversification. An increase in the incomes of the household heads enables them to purchase foodstuffs from the markets to feed their families without necessarily expanding their cocoa farms to generate higher incomes.

## Summary, conclusions and recommendations

This study employed both quantitative and qualitative research methods to examine the dietary diversity and food security among male and female-headed cocoa households. The study rejected the null hypothesis because there was a significant relationship between food security status and the gender of the household head. Through this study, it was revealed that a higher percentage of male-headed households had high dietary diversity and were food secure compared to female-headed households. Also, roots/tubers, local grains, and vegetables happened to be the top three consumed foods, while organic meat, eggs, fruits, legumes/nuts, and milk products were the least consumed. Furthermore, April, June, and July were generally the food shortage or unavailability months. Moreover, the majority of the male and female-headed households reported having obtained their food from both the market and self-production. In addition, the study discovered that more of the male-headed households had increased food production status, high dietary diversity, and were food secure than female-headed households. Again, the study identified several themes, such as climate change/variability, poverty, large household size, unavailable lands, and gender stereotypes/ beliefs that emerged as influential in promoting low dietary diversity and food insecurity among male and female-headed households. Sex, total household income, and cropland conversion were the variables influencing household heads’ perception of the impact of cocoa expansion on crop diversification. The study concludes that male-headed households are more food secure and food diverse than female-headed households in the study area. Policymakers can use the findings to determine appropriate public policies in the battle against low dietary diversity and food insecurity in rural communities. Thus, the study recommends the following interventions to improve household dietary diversity and food security: implement nutrition education programs to raise awareness about the need for diverse diets and to provide practical information on how to incorporate a greater variety of food groups into their meals; putting in place gender-sensitive programs to empower women in cocoa producing communities; giving credit assistance to cocoa farming households, particularly those headed by women to address poverty; and encourage crop diversification. Future research could investigate how gender mainstreaming policies in agriculture have helped empower and improve the food security of female-headed households in Ghana.

### Electronic supplementary material

Below is the link to the electronic supplementary material.

Questionnaires

## Data Availability

For data protection purposes, the datasets that contain the personal information of the participants cannot be shared publicly. Other non-traceable data is available with the corresponding author based on a reasonable request.

## References

[1] Ochieng J (2017). Determinants of dietary diversity and the potential role of men in improving household nutrition in Tanzania. PLoS ONE.

[2] Cordero-Ahiman OV (2021). Factors that determine the dietary diversity score in rural households: the case of the Paute River Basin of Azuay Province, Ecuador. Int J Environ Res Public Health.

[3] Cordero Ahimán OV, Santellano E, Estrada, Garrido A, Colmenero (2017). Dietary diversity in rural households: the case of indigenous communities in Sierra Tarahumara, Mexico. J Food Nutr Res.

[4] Mallikarjuna K (2013). Food security and climate change. Int J Res Appl Nat Social Sci.

[5] Frempong RB, Annim SK (2017). Dietary diversity and child malnutrition in Ghana. Heliyon.

[6] Asiedu B, Nunoo F, Iddrisu S. Prospects and sustainability of aquaculture development in Ghana, West Africa. Volume 3. Cogent Food & Agriculture; 2017. p. 1349531. 1.

[7] Kenkhuis M. *Nutritional status among cocoa farming families and underlying causes in Ghana* Student Nutrition and Health at Wageningen University, intern at GAIN. Global Alliance for Improved Nutrition; 2016.

[8] Ajagun EO et al. *Cocoa eats the food: expansion of cocoa into food croplands in the Juabeso District, Ghana* Food Security, 2021: p. 1–20.

[9] Ashiagbor G (2020). Pixel-based and object-oriented approaches in segregating cocoa from forest in the Juabeso-Bia landscape of Ghana. Remote Sens Applications: Soc Environ.

[10] Batame M. Global Trade and Local Food Security: Mapping and Monitoring Cocoa Expansion and its impact on Household Food Security in the Bia West Disctrict, Ghana. University of Twente; 2023.

[11] Chegere MJ, Stage J (2020). Agricultural production diversity, dietary diversity and nutritional status: panel data evidence from Tanzania. World Dev.

[12] Heim A, Paksi A (2019). Low dietary diversity and its influencing factors among a San group in Namibia. BMC Res Notes.

[13] Oldewage-Theron W, Kruger R (2011). Dietary diversity and adequacy of women caregivers in a peri-urban informal settlement in South Africa. Nutrition.

[14] Kadiyala S, Rawat R (2013). Food access and diet quality independently predict nutritional status among people living with HIV in Uganda. Public Health Nutr.

[15] Isabirye N (2020). Dietary diversity and associated factors among adolescents in eastern Uganda: a cross-sectional study. BMC Public Health.

[16] Kennedy G, Ballard T, Dop MC. Guidelines for measuring household and individual dietary diversity. Food and Agriculture Organization of the United Nations; 2011.

[17] Taruvinga A, Muchenje V, Mushunje A (2013). Determinants of rural household dietary diversity: the case of Amatole and Nyandeni districts, South Africa. Int J Dev Sustain.

[18] Powell B (2017). The determinants of dietary diversity and nutrition: ethnonutrition knowledge of local people in the East Usambara Mountains, Tanzania. J Ethnobiol Ethnomed.

[19] Idowu O, Olusayo AO. *Dietary diversity status of rural households in Nigeria: A gendered perspective* Dietary diversity status of rural households in Nigeria: a gendered perspective, 2019: p. 613–636.

[20] Koppmair S, Kassie M, Qaim M (2017). Farm production, market access and dietary diversity in Malawi. Public Health Nutr.

[21] Dei Antwi K, Lyford CP, Nartey RY. *Analysis of food security among cocoa producing households in Ghana*. J Agric Sustain, 2018. 11(2).

[22] Mehraban N, Ickowitz A (2021). Dietary diversity of rural Indonesian households declines over time with agricultural production diversity even as incomes rise. Global Food Secur.

[23] Codjoe SNA, Okutu D, Abu M (2016). Urban household characteristics and dietary diversity: an analysis of food security in Accra, Ghana. FoodNutr Bull.

[24] Kumar I, Gautam M (2022). Determinants of Dietary Diversity score for the rural households of Uttar Prades State. Int J Food Nutr Diet.

[25] Aidoo R, Mensah JO, Tuffour T. *Determinants of household food security in the Sekyere-Afram Plains district of Ghana*. Eur Sci J, 2013. 9(21).

[26] Annim SK, Frempong RB (2018). Effects of access to credit and income on dietary diversity in Ghana. Food Secur.

[27] Kiewisch E (2015). Looking within the household: a study on gender, food security, and resilience in cocoa-growing communities. Gend Dev.

[28] Jebessa GM, Sima AD, Wondimagegnehu BA (2019). Determinants of household dietary diversity in yayu biosphere reserve, Southwest Ethiopia. Ethiop J Sci Technol.

[29] Mekuria G, Wubneh Y, Tewabe T (2017). Household dietary diversity and associated factors among residents of finote selam town, North West Ethiopia: a cross sectional study. BMC Nutr.

[30] Dube L, Guveya E (2016). Factors influencing smallholder crop diversification: a case study of Manicaland and Masvingo provinces in Zimbabwe. Int J Reg Dev.

[31] Meinzen-Dick RS et al. *Gender, assets, and agricultural development programs: A conceptual framework* CAPRi Working Paper, 2011.

[32] Kanter R (2015). A conceptual framework for understanding the impacts of agriculture and food system policies on nutrition and health. Food Secur.

[33] Pieters H, Guariso A, Vandeplas A. Concept Framew Anal Determinants food Nutr Secur. 2013.

[34] Bank G. *SECTOR INDUSTRY ANALYSIS 2022 COCOA SECTOR REPORT* 2022.

[35] Ghana Statistical S. *2010 Population and Housing Census; Juaboso District Analytical Report*. 2014.

[36] Ashiagbor G (2022). Monitoring cocoa-driven deforestation: the contexts of encroachment and land use policy implications for deforestation free cocoa supply chains in Ghana. Appl Geogr.

[37] Wheeldon J (2010). Mapping mixed methods research: methods, measures, and meaning. J Mixed Methods Res.

[38] Teye JK (2012). Benefits, challenges, and dynamism of positionalities associated with mixed methods research in developing countries: evidence from Ghana. J Mixed Methods Res.

[39] Miller RL, Brewer JD. The AZ of social research: a dictionary of key social science research concepts. Sage; 2003.

[40] Sultana S (2021). Prevalence and factors associated with depression among the mothers of school-going children in Dhaka city, Bangladesh: a multi stage sampling-based study. Heliyon.

[41] Taylor B. *Assessing the food security status and production constraints of cocoa farming households in the Ashanti region*. 2017, Thesis of master of philosophy, University of Ghana, Legon, Ghana.

[42] Ajagun EO (2022). Cocoa eats the food: expansion of cocoa into food croplands in the Juabeso District, Ghana. Food Secur.

[43] Aneani F (2012). Adoption of some cocoa production technologies by cocoa farmers in Ghana. Sustainable Agric Res.

[44] Limb M, Dwyer C. Qualitative methodologies for geographers: issues and debates. Oxford University Press; 2001.

[45] Al-Zabir A (2021). Food security status of farming households in Bangladesh: a comparison of recipients and non-receivers of institutional support. Afr J Sci Technol Innov Dev.

[46] Legwegoh AF, Hovorka AJ (2013). Assessing food insecurity in Botswana: the case of Gaborone. Dev Pract.

[47] Hernandez A. *Why Are Fruits & Vegetables Important?* 2018 [cited 2023 27– 02]; Available from: https://healthyeating.sfgate.com/fruits-vegetables-important-4703.html.

[48] Anderman TL (2014). Synergies and tradeoffs between cash crop production and food security: a case study in rural Ghana. Food Secur.

