# Supplementary material for: Unlocking gender dynamics in food and nutrition security in Ghana: assessing dietary diversity, food security, and crop diversification among cocoa household heads in the Juaboso-Bia cocoa landscape
Source: BMC Public Health. 2024 Apr 8;24:985. doi: 10.1186/s12889-024-18204-7 (PMC11003139; doi:10.1186/s12889-024-18204-7)
Supplement: Supplementary file 1 — Questionnaires [file 12889_2024_18204_MOESM1_ESM.docx]

**Appendice (Questionnaire)**

**Section A: Personal and Socio-Economic Information**

I would begin by asking about the head of your household. By household, I mean you and all the people (including children if any) with whom you eat from the same pot; and share the same accommodation and other resources like money, land, and equipment. Please indicate your answer by ticking [ √ ] in the bracket.

1. Sex of household head: 1. Male [ ] 2. Female [ ]

2. Age of household head: ……………………………..

3. What is the level of education attained by the household head?

1. Primary [ ] 2. JHS/Form 4 [ ] 3. SSS/O or A level [ ] 4. Tertiary [ ]

5. No formal education [ ]

4. What is the household head’s marital status?

1. Married [ ] 2. Single [ ] 3. Divorced [ ] 4. Widow/widower [ ]

4. What is your household size? …………………………….

5. How many of your children are working? ……………………………….

5b. What is the household’s dependency ratio? …………….

6. Do you belong to any cocoa cooperative group? 1. Yes [ ] 2. No [ ]

7. Was some or all of the land (currently used for cocoa production) previously used as cropland? 1. Yes [ ] 2. No [ ]

8. Did the household head have access to extension officers? 1. Yes [ ] 2. No [ ]

9. How much income did your household earn from cocoa proceeds in 2022? (GH¢) ………

10. What was your total household income in 2022? 1. less than 10,000 [ ] 2. Between 10,000 and 30,000 [ ] 3. Above 30,000 [ ]

**Section B: Household Food Security Assessment**

11. Does your household engage in food crop farming? 1. Yes [ ] 2. No [ ]

12. What is your household’s major source of staple foods? 1. Self-production [ ]

2. Market [ ] 3. Self-production and market [ ]

13. What has been the general trend in your household’s food production in 2022?

1. Decreasing [ ] 2. Increasing [ ] 3. No noticeable change [ ]

14. Did your household experience food shortages in 2022?

1. Yes [ ] 2. No [ ]

15. If yes, in which month(s) did your household experience this shortage/unavailability?

| **Month** | Jan | Feb | Mar | Apr | May | Jun | Jul | Aug | Sep | Oct | Nov | Dec |
| --- | --- | --- | --- | --- | --- | --- | --- | --- | --- | --- | --- | --- |
| **Tick ✓** |  |  |  |  |  |  |  |  |  |  |  |  |

16. Which food group(s) did the household consume in the past 24-hours?

To Enumerator: the table below is the dietary diversity score, in which he or she will score a household one (1) if the household ate any food from each of the food groups, otherwise zero (0).

Household dietary diversity score

| **Food groups** | **Points (Yes = “1”; No = “0”)** |
| --- | --- |
| 1. Any bread, rice, noodles, biscuits, or any other foods made from millet, sorghum, maize, rice, wheat, or any other locally available grain |  |
| 2. Any potatoes, yams, manioc, cassava, or any other foods made from roots or  tubers |  |
| 3. Any vegetables |  |
| 4. Any fruits; mango, orange, pineapple, etc |  |
| 5. Any beef, pork, lamb, goat, rabbit, wild game, chicken, duck, other birds,  liver kidney, heart, or other organ meats |  |
| 6. Any eggs |  |
| 7. Any fresh, dried fish or shellfish |  |
| 8. Any legumes; beans, peas, lentils, or nuts |  |
| 9. Any cheese, yogurt, milk, or other milk products |  |
| 10. Any foods made with oil, fat, or butter |  |
| 11. Any sugar or honey |  |
| 12. Any other foods such as spices, condiments, coffee, or tea |  |
| **Total Points (12)** |  |

Source: (Taruvinga et al., 2013)

**In-depth interview guidelines**

1. Do you live in this district?

2. Which community do you live in?

3. How long have you been living in this community?

4. How long have you been farming cocoa in this community?

5. What are the major food crops grown in this district?

6. How is food farming being managed?

7. Have you changed your cropland to cocoa? If yes, has it affected your food security and dietary diversity?

8. What can you say about your food production over the years?

9. Where do you get your food to feed your family, and why that source?

10. What does your household usually consume in a day, and why?

11. What is your experience with food shortage or unavailability? * probe coping strategies

12. What is your view on cocoa expansion's impact on crop diversification?

13. What are the reasons for low dietary diversity and food insecurity among headed by men and women?

14. Have you experienced any gender stereotypes or socio-cultural barriers to the food you consumed, and why?

15. From your experience, how can cocoa farmers balance the cultivation of cocoa and food crop production, as well as ensure forest security?
